# Facile and cost-effective production of microscale PDMS architectures using a combined micromilling-replica moulding (*μ*Mi-REM) technique

**DOI:** 10.1007/s10544-015-0027-x

**Published:** 2016-01-08

**Authors:** Dario Carugo, Jeong Yu Lee, Anne Pora, Richard J. Browning, Lorenzo Capretto, Claudio Nastruzzi, Eleanor Stride

**Affiliations:** BUBBL, Institute of Biomedical Engineering, Department of Engineering Science, University of Oxford, Old Road Campus Research Building, Oxford, OX3 7DQ UK; School of Pharmacy, University College London (UCL), London, WC1E 6BT UK; Department of Life Sciences and Biotechnology, University of Ferrara, I-44121 Ferrara, Italy

**Keywords:** Microfabrication, Microfluidic, Microchannel, Micromilling, Pmma, Replica moulding, Pdms, Emulsions, Microbubbles

## Abstract

We describe a cost-effective and simple method to fabricate PDMS-based microfluidic devices by combining micromilling with replica moulding technology. It relies on the following steps: (i) microchannels are milled in a block of acrylic; (ii) low-cost epoxy adhesive resin is poured over the milled acrylic block and allowed to cure; (iii) the solidified resin layer is peeled off the acrylic block and used as a mould for transferring the microchannel architecture onto a PDMS layer; finally (iv) the PDMS layer is plasma bonded to a glass surface. With this method, microscale architectures can be fabricated without the need for advanced technological equipment or laborious and time-consuming intermediate procedures. In this manuscript, we describe and validate the microfabrication procedure, and we illustrate its applicability to emulsion and microbubble production.

**Electronic supplementary material:**

The online version of this article (doi:10.1007/s10544-015-0027-x) contains supplementary material, which is available to authorized users.

## Introduction

A plethora of fabrication methods has been developed in recent years to construct micro- and nano-scale architectures for fluid manipulation (Quake and Scherer 2000; Madou 2002). The selection of a specific fabrication technique for a given application usually involves a compromise between: (i) constraints imposed by the properties of the materials used, (ii) technological complexity and (iii) economic factors. Table 1 summarises the microfabrication methods that have been adopted most widely within the microfluidic community, together with the materials commonly used.

**Table 1:** Summary of the most commonly used microfabrication methods and corresponding materials. PDMS = poly(dimethylsiloxane); PMMA = poly(methylmethacrylate); PC = polycarbonate; PP = polypropylene; PET = poly(ethylene terephthalate); COC = cyclic olefin copolymer

Fabrication Method	Materials
Soft lithography	*PDMS* (Eddings et al. 2008) *Thiol-ene formulations* (Ashley et al. 2011; Carlborg et al. 2011) *Hydrogels* (Chung et al. 2012)
Wet etching	*Silicon* (Tsujino and Matsumura 2007) *Glass* (i.e*.*, *fused-silica*, *Pyrex*, *soda-lime*) (Grosse et al. 2001; Lin et al. 2001; Capretto et al. 2011)
Dry Etching	*PDMS* (Oh 2008) *Glass* (Park et al. 2005)
3D Printing	*PP* (Kitson et al. 2012) *Acrylate-based polymers*/*resins* (Erkal et al. 2014)
Hot embossing	*PMMA* (Lee et al. 2001; Qi et al. 2002; Narasimhan and Papautsky 2004) *PC* (Becker and Heim 2000) *PET* (Li et al. 2008) *COC* (Jeon et al. 2011)
Micromilling	*PMMA* (Carugo et al. 2012) *COC* (Ogilvie et al. 2010) *Ceramic* (Carugo et al. 2014)
Laser micromachining	*PMMA* (Klank et al. 2002) *Glass* (Liao et al. 2012)

Among the different techniques, soft lithography has attracted considerable interest given its potential for constructing a variety of two- and three-dimensional architectures, with materials and surface properties that are compatible with multiple applications (Qin et al. 2010). Replica moulding (REM) is a common patterning technique used in soft lithography and is particularly useful as a means of fabricating poly(dimethylsiloxane) (PDMS)-based microfluidic devices (Qin et al. 2010). Notably, PDMS possesses a range of unique features compared to other materials reported in Table 1, which makes it an attractive choice particularly for constructing microfluidic systems designed for biomedical applications (Mata et al. 2005). These properties include: (i) optical transparency (Schneider et al. 2009), (ii) gas permeability (Merkel et al. 2000) (Bose et al. 2012), (iii) biocompatibility (Borenstein et al. 2002; Wang et al. 2007; Borenstein et al. 2010), (iv) ability to conform to a surface (Qin et al. 2010), (v) intrinsic hydrophobicity and ease of surface treatment (i.e., by exposure to oxygen plasma) (Eddings et al. 2008), and (vi) adequate elasticity for development into microscale valves and fluid actuators (Unger et al. 2000). Moreover, PDMS has been recently employed to develop acoustically-transparent interfaces in applications combining microfluidics with ultrasound fields, given that its characteristic acoustic impedance is close to that of aqueous solutions (Leibacher et al. 2014; Carugo et al. 2015).

**Fig. 1:**
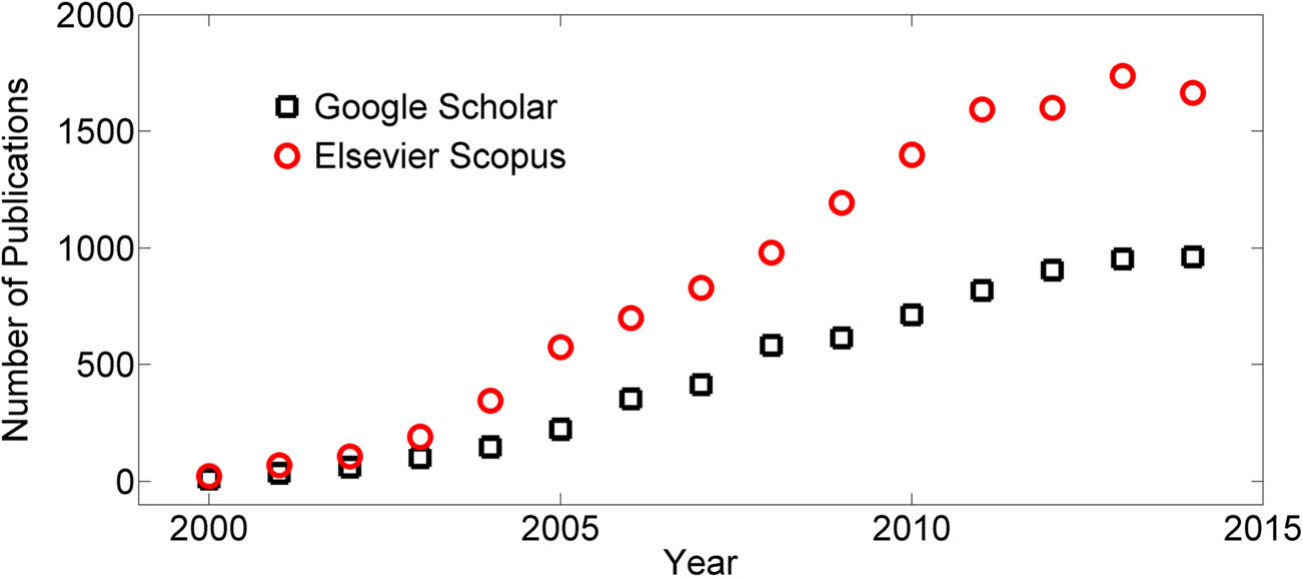
Temporal evolution of the number of scientific publications containing the keywords “PDMS + microfluidic” from Google Scholar (*empty black squares*) and Elsevier Scopus® (*empty red circles*) from 2000 to 2014

The popularity of PDMS in microfluidic applications is manifest in the growing number of publications that have appeared on scientific databases in the last 15 years, i.e., from 2000 to 2014 (see Fig. 1 for publications containing the keywords “PDMS + microfluidic”). Between 2000 and 2005 there was an exponential increase, most likely due to the adoption of soft lithography (Duffy et al. 1998), followed by an approximately linear increase until 2012. However, the volume of publications has remained virtually invariant in the last three years (i.e., from 2012 to 2014, see Fig. 1). This recent reduction in publication rate suggests that further efforts should be focused on developing microfabrication routes capable of satisfying the needs of the growing microfluidic and microtechnology research communities (Sackmann et al. 2014), and particularly in those expanding, low-resource settings for which traditional fabrication approaches may be technically and economically prohibitive.

The most popular technique for producing PDMS-based microfluidic devices by REM relies on the following steps: (i) a computer assisted design (CAD) of the microchannel geometry is generated; (ii) the geometry is printed on a chrome photomask; (iii) a positive mould (or master) of the geometry is generated by photolithography, i.e. a substrate (usually SU-8) is aligned with the photomask and exposed to ultraviolet (UV) light; (iv) liquid PDMS is introduced into the master and allowed to solidify; and (v) the PDMS layer containing the microchannel architecture is reversibly or irreversibly bonded to another surface layer (usually glass or PDMS).

Despite its widespread usage, the adoption of this technique in some laboratory settings may be limited by: (i) the need for clean room facilities, (ii) lack of adequate instrumentation, including spin coaters for generating the SU-8 substrate or photomask aligner and appropriate UV source for transferring the geometry to the SU-8 substrate, and (iii) cost of materials, particularly of photomasks (if these cannot be produced in house) and SU-8. Thus, as the range of applications for microfluidic devices grows, there is an increasing need for the development of less laborious and cheaper methods to construct PDMS-based microfluidic architectures. For this reason, several alternative replica moulding techniques have been proposed in recent years, including those utilising easy-to-fabricate moulds produced via 3D printing, wax deposition or micromilling.

The use of wax moulds represents an attractive, cost-effective and rapid route to fabricate PDMS microchannel architectures (Kaigala et al. 2007), however it suffers from the need for specialised wax printers. Alternative, less expensive wax deposition methods have been proposed (for example, drop-on-demand) (Hou et al. 2014), but may result in undesired surface patterns (referred to as “fluctuating surfaces”). In addition, microchannels fabricated with this technique have minimum width of ~200 μm, and it is non-trivial to achieve fine control over the microchannel cross-sectional geometry. Furthermore, in some cases wax moulds are not re-usable for multiple moulding iterations (Hou et al. 2014).

Greater flexibility over the three-dimensional geometrical characteristics of the microfluidic architectures can be achieved by using 3D printed moulds (McDonald et al. 2002; Comina et al. 2013; Chan et al. 2015). This approach however is again limited in terms of the minimum channel size achievable (high resolution 3D printers are available on the market, but at significant cost), and by surface patterns resulting from the layer-by-layer material deposition typical of 3D printers which may cause undesired features to appear on the inner PDMS surfaces of the finished product. Furthermore, this method often requires intermediate, time-consuming thermal and chemical treatments of the 3D printed master which, if left untreated, would inhibit PDMS polymerisation at the master-PDMS interface (Chung et al. 2012; Comina et al. 2013). As for methods based on wax moulds, 3D printed masters may be re-used only for a limited number of moulding cycles (approximately 3–4 in Comina et al. 2013 and McDonald et al. 2002) due to mechanical failure of the smallest, more delicate features.

An alternative method was proposed by Wilson and co-authors in 2011, which combines mechanical milling with soft lithography for producing PDMS circular channels, by introducing an intermediate moulding step (Wilson et al. 2011). This approach has considerable potential advantages but the selection of materials and implementation of the methods described in Wilson et al., severely limits the minimum channel dimension achievable (only channels with ~1 mm diameter have been reported in the literature using this method). Moreover, the method as described in the paper, requires the use of highly specialised machining tools (a combined Miniature Machine Tool and optical system) in addition to intermediate surface passivation steps, and the use of laborious techniques, such as polishing with alumina particles, chemical etching or powder blasting to reduce residual surface roughness. These factors may have hindered the adoption of this method in the wider microfluidic and lab-on-a-chip communities.

The use of thermally aged PDMS as an intermediate moulding material has also been demonstrated for constructing PDMS microfluidic devices starting from a microchannel architecture milled into an acrylate layer (Ziółkowska et al. 2011). This method, referred to as double casting prototyping, has proved to be successful in fabricating small microchannel features (i.e., ~100 μm) at relatively low-cost and without the need for chemical additives. Curing and hardening of the PDMS master is however a relatively time-consuming procedure (i.e., 3 h curing + 48 h hardening), and thermally aged masters may be used for a limited number of fabrication cycles (i.e., up to 20 cycles) (Kwapiszewska et al. 2014).

In this paper we attempt to address the above limitations to create a facile method (*μ*Mi-REM) for constructing PDMS-based microfluidic devices with channel sizes suitable for a wide range of applications and that does not require specialised equipment or intermediate chemical functionalisation steps.

## Experimental

### Chemicals

Poly(dimethylsiloxane) (Sylgard® 184) was purchased from Dow Corning Corporation (Michigan, USA), and poly(methyl methacrylate) from theplasticshop.co.UK (Coventry, UK). Epoxy adhesive (Yellow Dual Cartridge) was purchased from RS Components Ltd. (Corby, UK). Poly(lactic-co-glycolic acid), Pluronic® F127, dichloromethane, polyoxyethylene (40) stearate, trichloro-(1 H, 1 H, 2 H, 2 H-perfluorooctyl)-silane, and Evans blue dye were purchased from Sigma Aldrich (Gillingham, UK). Phosphate buffered saline was purchased from Life Technologies (Thermo Fisher Scientific Inc., Massachusetts, USA) and 1,2-distearoyl-sn-glycero-3-phosphocholine from Avanti Polar Lipids (Alabama, USA). Perfluorohexane was purchased from Apollo Scientific Ltd. (Stockport, UK). Nitrogen (N_2_) gas was provided by The BOC Group plc (Guildford, UK).

### Microdevice fabrication using *μ*Mi-REM

**Fig. 2:**
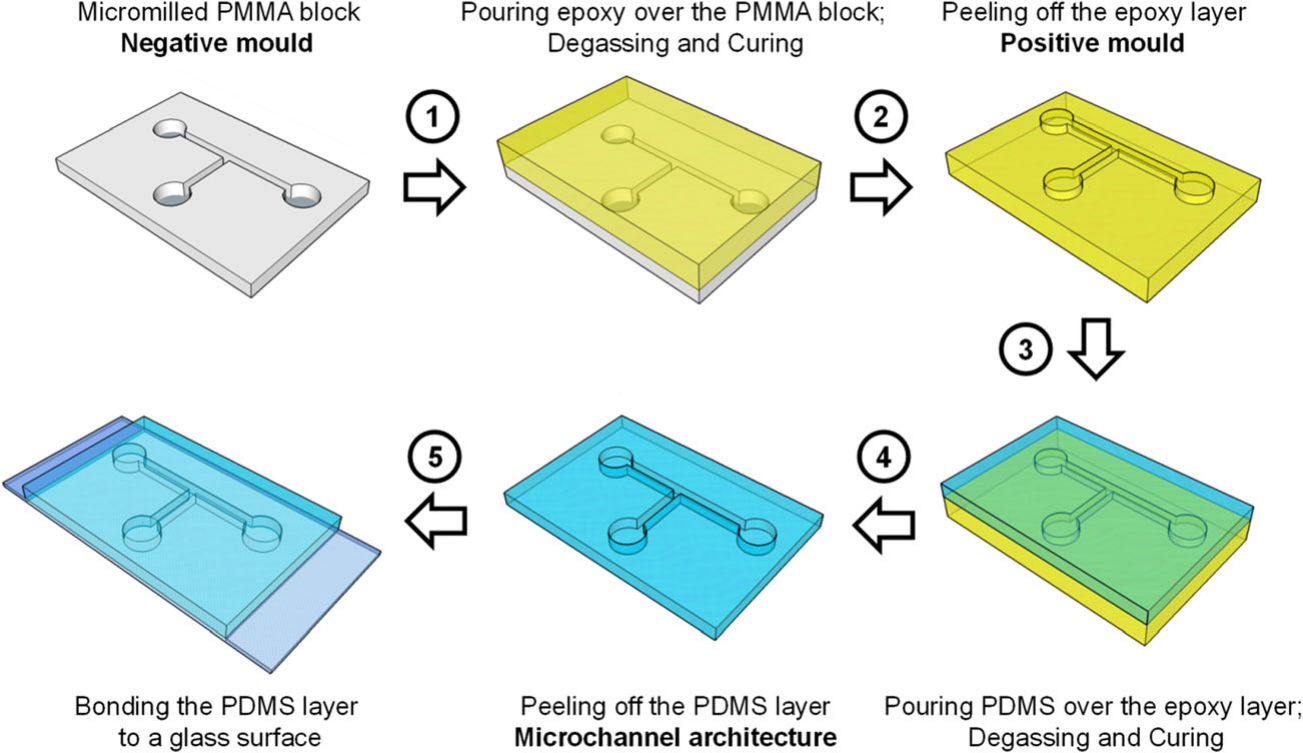
Microdevice fabrication by combined micromilling and replica moulding (*μ*Mi-REM)

The fabrication technique comprises the following steps (Fig. 2):

(i) CAD of the microdevice architecture. In the example shown here SolidWorks 2012 (Dassault Systèmes SOLIDWORKS Corp., Velizy Villacoublay, France) was used.
(ii) Milling of the geometry on to a block of poly(methyl methacrylate) (PMMA) using a milling machine (XYZ 3000, XYZ Machine Tools, Tiverton, UK). The block was employed as a negative mould^1^ in the subsequent fabrication steps.
(iii) Epoxy casting of the mould. A bi-component, low-cost liquid epoxy adhesive was poured over the PMMA block. A 1:1 ratio (by weight) between the two components was identified as the optimal one for this application. In order to prevent the epoxy from leaking out from the PMMA block, adhesive tape was applied to the lateral edges of the block. Approximately 6 g of epoxy were needed to form a ~ 4 mm thick layer over a 4 mm × 4 mm surface.
(iv) Degassing by placing the device in a vacuum chamber for 5 min. This step is needed to prevent air bubbles from being trapped within the microchannels prior to epoxy solidification. Bubbles will generate invaginations within the solidified epoxy block, potentially compromising the usability of the device (see Supplementary Figure S1). It is difficult to achieve complete degassing of the epoxy layer during this step (due to the increasing viscosity of epoxy during solidification), however this method has proved to be successful in removing bubbles from the microchannels (i.e., in the *z*-direction), at a distance >1 mm from the PMMA surface (see Supplementary Figure S1).
(v) Solidification of the epoxy which at normal room temperature (~ 21 °C) requires ~100 min.
(vi) Removal of the epoxy layer from the PMMA block using a surgical scalpel. This layer contains the microdevice architecture and is the positive mould (or master) for the remaining steps.
(vii) Casting of the PDMS device. In this example, liquid PDMS (at a curing agent:monomer ratio of 1:10 *w*/*w*) was poured over the epoxy layer, degassed in a vacuum chamber for approximately 20 min, and cured overnight at ambient temperature. Higher curing temperatures (i.e., up to 40 °C or higher) may be employed to reduce the curing time; however, attention must be paid to the release of entrapped air bubbles from the epoxy layer into the PDMS due to potential softening of epoxy resin at these higher temperatures.
(viii) Removal of the solidified PDMS layer containing the microchannels from the epoxy layer again using a surgical scalpel. Here the device was then bonded to a 1 mm thick glass layer (Corning® microscope slides, Sigma Aldrich, Gillingham, UK) by plasma treatment (plasma cleaner ATTO, Diener electronic GmbH, Ebhausen, Germany). The glass was cleaned with soap and deionised water (Merck Millipore, Billerica, USA), and dried with lint-free wipes (KIMTECH, Kimberly-Clark Worldwide, Inc., West Malling UK) prior to the plasma treatment. A photograph of a finished microdevice is shown in Fig. 4d.

In order to connect the microdevice with tubing for fluid introduction/discharge (i.e., macro- to micro-fluidic interfacing), two alternative methods were adopted: (method I) holes were created through the PDMS block using a blunt needle (BD, Becton, Dickinson and Company, New Jersey, USA) and 1/16″ outer diameter (OD) polytetrafluoroethylene (PTFE) tubes (Sigma Aldrich, Gillingham, UK) were inserted within the holes; and (method II) 1 cm long segments of 1/16″ OD polyether ether ketone (PEEK) rods (IDEX Corporation, Illinois, USA) were glued onto the epoxy layer in correspondence to the inlet/outlet reservoirs, using a low-cost, solvent-free glue (Pritt, Henkel Ltd., Herts, UK). The rods were removed after PDMS curing, and segments of 3/32″ OD Tygon® tubing (Cole-Parmer Instrument Co. Ltd., London, UK) were inserted in the formed holes and used as connectors with 1/16″ OD tubing (see Supplementary Figure S2). Depending on the solvents used in the experiments, 3/32″ OD Tygon® tubing could also be directly used as inlet/outlet lines in this method without the need for additional connection elements (as shown in Fig. 4d), providing a rapid and convenient strategy for fluid injection and discharge.

### SEM imaging of microchannels

**Fig. 3:**
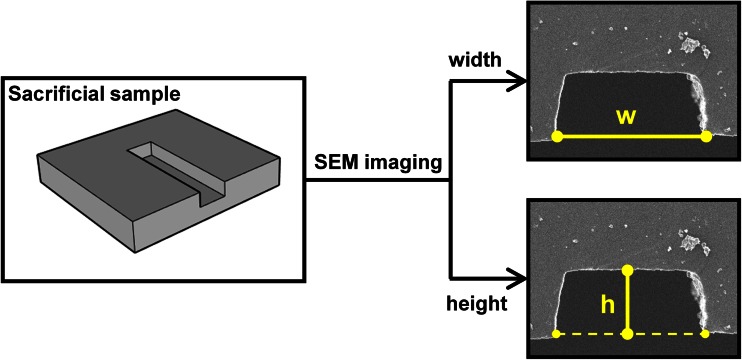
Imaging of channel cross-section using scanning electron microscopy (SEM) and measurement of channel width (w) and height (h) from the acquired microscope images. These were quantified for each moulding step. For the representative PDMS microchannel reported here, h = 117.8 μm and w = 272.6 μm

In order to validate the fabrication technique and evaluate the effect of sequential moulding steps on microchannel geometry, Scanning Electron Microscopy (SEM) imaging was carried out. A sacrificial microdevice was constructed for this purpose (*n* = 3). The microdevice consisted of a straight microchannel architecture, with nominal channel width and height of 254 μm and 100 μm, respectively (Fig. 3). A cut through the micromilled PMMA block (negative mould), the epoxy layer (positive mould) and the PDMS layer was performed, so that the cross-sectional geometry of the microchannel could be revealed and visualised by SEM (Fig. 3). Samples were coated with a 8 nm thick layer of gold using a dual carbon/sputter coater (Q150R, Quorum Technologies, Lewes, UK) and placed on the SEM stage for imaging (JSM-6390, Jeol, Tokyo, Japan). The microchannel’s width and height were measured from the acquired microscope images using ImageJ (NIH, USA) (as in Fig. 3). Representative SEM images of PDMS microchannels fabricated by *μ*Mi-REM are also illustrated in Fig. 5a-d.

### Application of *μ*Mi-REM technology

**Fig. 4:**
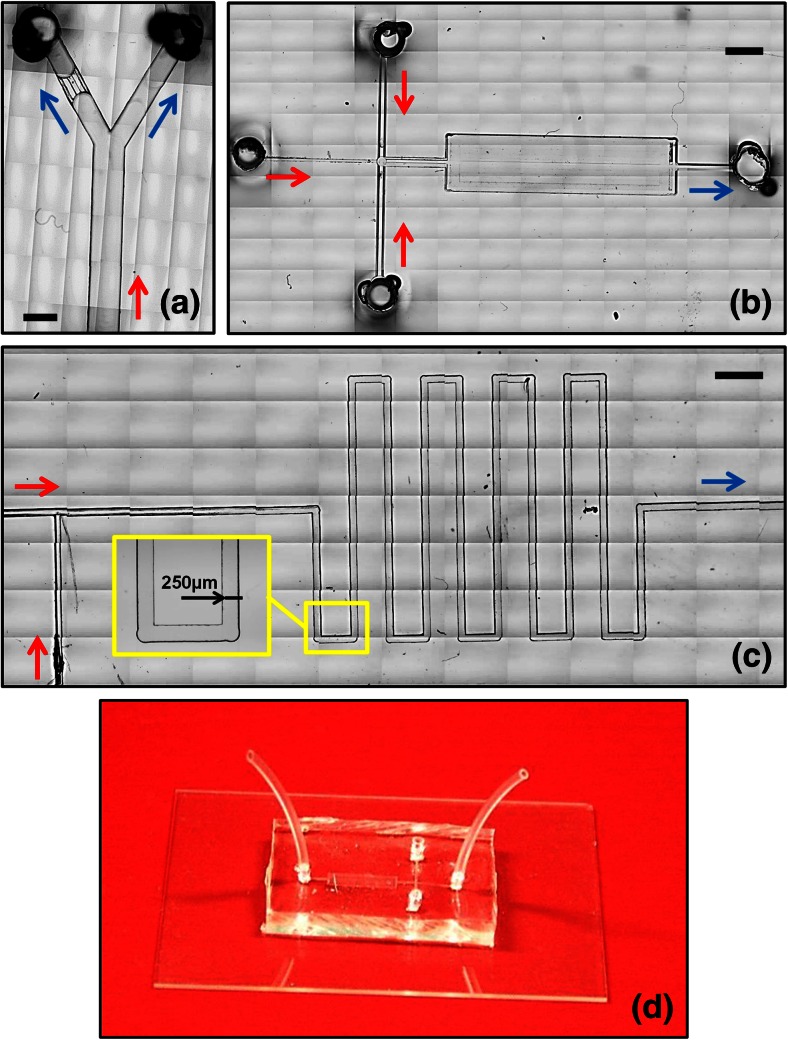
**a**-**c** Bright-field microscope images of microfluidic devices fabricated using *μ*Mi-REM. The images were created from a stack of multiple microscope acquisitions over a large surface area of the device, acquired using a Nikon ECLIPSE Ti inverted microscope (Nikon Corporation, Tokyo, Japan) coupled with a charge-coupled device (CCD) camera (Digital sight Ds-Fi1, Nikon Corporation, Tokyo, Japan). The red and blue arrows indicate the inflow and outflow lines, respectively. The inlet and outlet ports in (**a**) and (**b**) were fabricated using the method reported in Supplementary Section S2. **a** Single bifurcation architecture, with a ‘micro-filter’ structure on the left daughter branch (microchannels are 500 μm deep). **b** Cross-flow architecture followed by a micro-chamber for visualization purposes (microchannels are 50 μm deep). **c** T-junction architecture followed by a serpentine-like structure. A magnified view of a serpentine section is shown in the inset (microchannels are 50 μm deep). **d** Finalised microfluidic device, with the PDMS layer containing the microchannel architecture plasma bonded to a 1 mm thick glass layer. Inlet and outlet tubing can be directly connected to the inlet/outlet ports of the device (through pre-formed reservoirs), without the need for additional connection elements. Scale bars in (**a**), (**b**), (**c**) are 500 μm, 2 mm, and 1 mm, respectively.

In order to assess the performance of microdevices fabricated using this technique, a set of devices was constructed and employed to produce: (i) encapsulated liquid droplets of phosphate buffered saline (PBS) -*in*- poly(lactic-*co*-glycolic acid) (PLGA) and (ii) gas microbubbles stabilised by a phospholipid shell. The device consisted of a ‘T-junction’ architecture (of the type illustrated in Fig. 4c) with two inlet (IN1 and IN2) and one outlet (OUT) flow lines. Syringe pumps (World Precision Instruments Inc., Florida, USA) were employed to control the fluid flow through the inlet lines, whilst the gas employed for microbubble production was provided by a pressurised cylinder, and the pressure measured using a digital manometer (2023P Digitron, Elektron Technology, Cambridge, UK). The chemicals and operating conditions used for these experiments are reported in Table 2.

**Table 2:** Processing conditions and chemicals used for the production of PBS-*in*-PLGA droplets and phospholipid-shelled microbubbles, using the microfluidic architecture illustrated in Fig. 6a. PBS = phosphate buffered saline; PLGA = poly(lactic-co-glycolic acid); DCM = dichloromethane; DSPC =1,2-Distearoyl-sn-glycero-3-phosphocholine; PEG40 = polyoxyethylene (40) stearate

PBS-in-PLGA droplets		
Inlet Line	Chemical	Processing Conditions
IN1	PBS + Pluronic® F127 (1 wt%)	1.2 mL/h
IN2	PLGA (6 wt% in DCM)	6.5 mL/h
Phospholipid-shelled microbubbles		
IN1	Nitrogen (N_2_)	Pressure = 36 kPa
IN2	DSPC:PEG40 (9:1 M ratio) in deionised water	30 mL/h

For the production of PBS-in-PLGA emulsions, the wettability of the inner surfaces of the device was altered via functionalisation using hydrophobic trichloro-(1 H, 1 H, 2 H, 2 H-perfluorooctyl)-silane, allowing for droplet break-up at the T-junction to occur (see Supplementary Video S3) (Nisisako et al. 2005). For this purpose, a solution of silane in perfluorohexane (PFH, 5 % by volume) was injected into the device and incubated for 1 h within a laminar flow hood, while keeping the inlet and outlet lines of the device closed. The device was subsequently rinsed with PFH, ethanol and finally filtered deionised water.

## Results and discussion

**Fig. 5:**
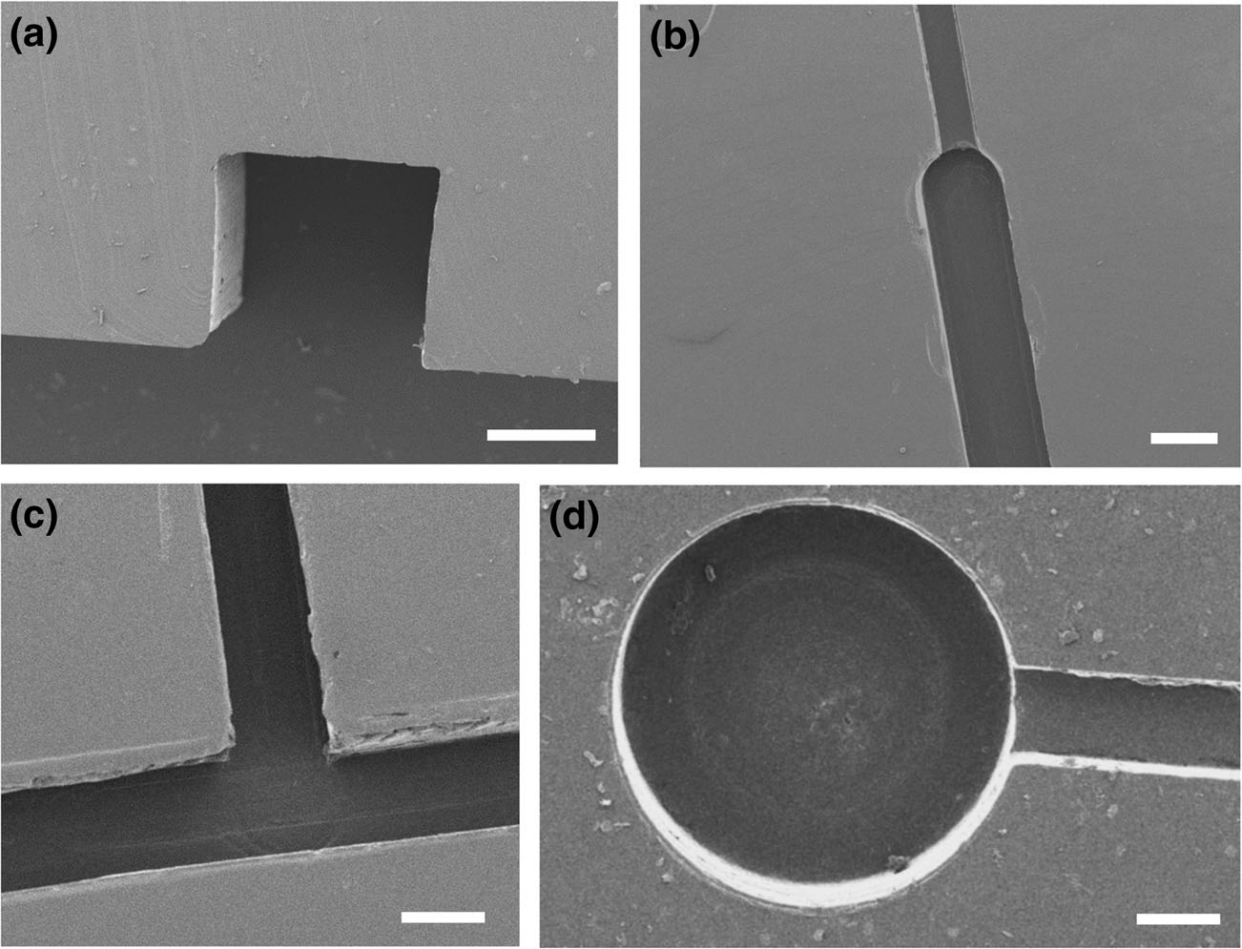
Representative SEM images of PDMS microchannels fabricated using *μ*Mi-REM. Scale bars are equal to 250 μm (**a**), 125 μm (**b**), 500 μm (**c**), and 200 μm (**d**)

Figures 4a-c show optical photomicrographs of different PDMS microfluidic architectures, fabricated using *μ*Mi-REM. A photograph of a finalised microfluidic device (of the type illustrated in Fig. 4b) is shown in Fig. 4d, with the PDMS layer bonded to a 1 mm thick glass layer. Short sections of Tygon® tubing were directly inserted within pre-formed inlet/outlet reservoirs (see Supplementary Section S2), providing a rapid and efficient connection route. Further SEM characterisation of the PDMS microchannels is shown in Fig. 5a-d. The microscope images demonstrate the potential of the developed technique to construct a variety of microfluidic chips designed for different purposes, and with microchannel dimensions relevant to a wide range of microfluidic applications. The minimum channel width and depth achieved with this method were ~100 μm and ~50 μm, respectively.

Table 3 summarises the minimum channel width and height achievable with other replica moulding approaches (i.e., relying on either wax, 3D printed or micromilled moulds), compared to *μ*Mi-REM. Notably, the dimensions of the microchannels obtained using *μ*Mi-REM are an order of magnitude smaller than those reported by Wilson et al. using an analogous fabrication approach (Wilson et al. 2011), and are comparable to those obtainable from masters made of wax, 3D printed polymers/resins or thermally aged PDMS. Channel depths as low as 10 μm have been reported using wax moulding (Kaigala et al. 2007) but not without undesirable surface patterning. It is envisaged that channel depths <50 μm could be achieved with our method, although these have not been investigated in the present study.

**Table 3:** Minimum microchannel depth and width (in μm) achieved using replica moulding approaches (excluding photolithography-based methods). The dimensions refer specifically to microfluidic channels, and not to other micro-scale components (i.e., valves, actuators, etc.)

Fabrication method	Authors	Minimum channel depth (in μm)	Minimum channel width (in μm)
Wax moulds	Kaigala et al. (2007)	~ 10	~ 250
	Hou et al. (2014)	~ 200	~ 200
3D printed moulds	McDonald et al. (2002)	~ 250	~ 250
	Comina et al. (2013)	~ 50	~ 50
	Chan et al. (2015)	~ 300	~ 300
Micromilled moulds	Wilson et al. (2011)	~ 1000	~ 1000
	Ziółkowska et al. (2011)	~ 50	~ 100
	μMi-REM	~ 50	~ 100

Furthermore, although smoothing of the inner surfaces of the microchannels was not performed in this study, there was no noticeable, major alteration of the microchannel geometry due to surface roughness originating from the micromilling step (see Fig. 5a for a magnified view of microchannel cross-section). The choice of materials in our technique is such that facile, rapid and low-cost methods (e.g. surface exposure to chloroform vapours) can be employed to reduce the roughness of the PMMA mould to obtain optical quality microfluidic devices (Ogilvie et al. 2010). In contrast, smoothing of metal moulds as used in analogous fabrication approaches (Wilson et al. 2011), requires laborious polishing techniques, which may be particularly complex at the lengthscales relevant for microfluidic applications.

The intricacy of the microfluidic geometries produced by *μ*Mi-REM strongly depends on the technical capabilities of the milling machine used to construct the negative PMMA mould. In the present study, a conventional manual milling machine was used, offering excellent cost-effectiveness. Despite the simplicity of our approach however, microchannel architectures widely employed in the microfluidic and lab-on-a-chip communities could be constructed, such as networks, bifurcations, cross-flow, T-junction, and serpentine-like micro-structures (see Fig. 4), demonstrating superior flexibility over the obtainable microchannel geometry compared to other methods. Computer numerical control (CNC) milling machines could be employed to achieve even higher levels of geometrical complexity, including the possibility of fabricating three-dimensional and multi-level architectures, at higher spatial resolution (Guckenberger et al. 2015). These would be laborious to fabricate using conventional lithographic approaches (Sackmann et al. 2014).

Integration with macroscale equipment (i.e., micro-to-macro scale interfacing) in our method may also be easier to achieve, compared to traditional photolithography. For instance, the tubing connection approach identified as method II in Section 2.2 allows for direct interfacing of the inlet/outline tubing with the microfluidic channels, without resorting to intermediate connectors or the need for punching holes through the PDMS layer using blunt needles, which could potentially cause the release of debris within the microchannels and generate undesirable occlusions. Notably, by using this connection approach, we experienced a significant increase in the lifetime of the produced microfluidic devices.

As shown in Table 4, the microchannel geometry did not change significantly over the multiple moulding steps. A slight reduction in the average channel aspect ratio (defined as channel width/height) was observed in the first moulding step (i.e., from 2.49 to 2.25), but this increased again in the second moulding step (i.e., from 2.25 to 2.34). It should be noted that uncertainties in the measurement may have been caused by the procedure of microchannel slicing prior to SEM imaging (Fig. 3).

**Table 4:** Measured microchannel width and height after each moulding step. Values are reported as the mean average and standard deviation (*n* = 3). The nominal height and width correspond to 100 μm and 254 μm, respectively

Width (μm)		
PMMA	Epoxy resin	PDMS
268.8 ± 4.6	259.7 ± 2.1	263.5 ± 7.9
Height (μm)		
PMMA	Epoxy resin	PDMS
108.0 ± 4.5	115.7 ± 3.3	112.5 ± 8.6

**Fig. 6:**
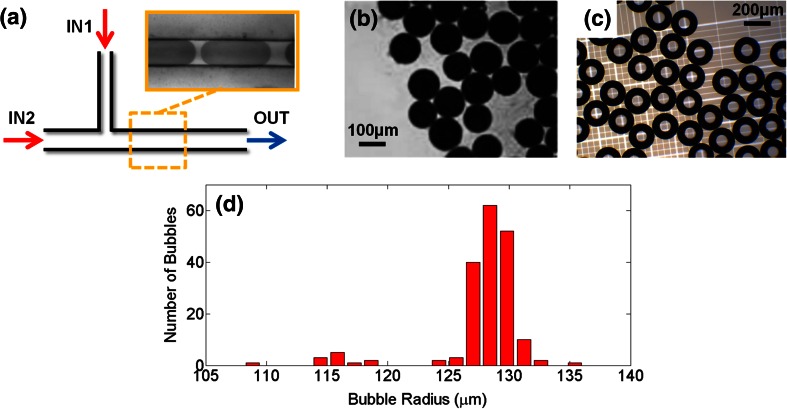
**(a)** Schematic of the ‘T-junction’ microfluidic device employed for the production of emulsions and microbubbles. Channel IN1 is 127 μm wide and 50 μm deep, whilst channels IN2 and OUT are 254 μm wide and 50 μm deep. The inset shows the formation of PBS-in-PLGA emulsions (stained using Evans blue) in a region of the device located after the T-junction. **b**-**c** Bright-field microscope images of PBS-in-PLGA emulsions (stained with Evans blue) (**b**) and phospholipid-shelled microbubbles (**c**). Images (magnification: 4×) were acquired using a Leica DM500 microscope (Leica Microsystems GmbH, Wetzlar, Germany) coupled with a CCD camera (MicroPublisher 3.3 RTV, QImaging, Surrey, Canada). **(d)** Representative size distribution of microbubbles obtained from a single experimental run (total number of counted bubbles =184). A population of bubbles with radius lower than 120 μm is present (corresponding to less than 8 % of the total bubble population) and is likely to be attributed to flow fluctuations originating from the stepped motor of the syringe pump

To demonstrate the utility of the proposed microfabrication technique, we employed microfluidic devices constructed by *μ*Mi-REM (Fig. 6a) to produce PBS-in-PLGA emulsions and phospholipid-shelled microbubbles. Representative microscope images of the emulsions/microbubbles obtained are shown in Fig. 6b and c, while Fig. 6d shows a representative size distribution plot of the obtained microbubbles from a single experimental run. The Supplementary Video S3 shows droplet formation within the microfluidic device.

## Conclusions

We present a facile and cost-effective method for fabricating microfluidic devices by combining micromilling (μMi) with replica moulding (REM) techniques: *μ*Mi-REM. With this method, PDMS-based microdevices can be constructed without the need for expensive equipment or chemicals. The method utilises conventional mechanical milling machines, such as those commonly available in mechanical workshops, and low-cost epoxy adhesive as the intermediate moulding material. Compared with analogous methods relying on micromilled metal moulds (Wilson et al. 2011), *μ*Mi-REM allows for the generation of significantly smaller microfluidic features and does not require intermediate chemical or physical surface treatment procedures. Double casting prototyping by thermal aging of PDMS is also suitable for fabricating small microchannel features without resorting to the use of chemical additives (Ziółkowska et al. 2011); however, the use of epoxy moulds in *μ*Mi-REM allows for rapid production of the master layer (i.e., ~100 min).

*μ*Mi-REM also provides superior control over the microchannel cross-sectional shape compared to other replica moulding approaches (i.e., those based on wax moulds) (Kaigala et al. 2007), by relying on the use of different micromilling tools (i.e., with square, ball or tapered end). Furthermore, it is suitable for the generation of multi-level three-dimensional geometries, and allows for facile integration with macroscale equipment. Notably, the estimated materials’ cost for a 5 × 5 cm^2^ device is approximately £3.50^2^ at the time of writing; which is a conservative prediction considering the potential for fabricating a large number of devices from a single epoxy mould. Epoxy has long-term stability, and masters currently in use have a lifetime greater than ~2 years at the time of writing. This is a significant advantage compared to other replica moulding techniques (McDonald et al. 2002; Comina et al. 2013) in which the positive master is either a sacrificial layer or it can be used only a limited number of times (Kwapiszewska et al. 2014). When compared to double casting prototyping using thermally aged PDMS, the upfront cost of materials is similar (~0.1 £/g of either epoxy or PDMS) but the increased re-usability of epoxy masters makes *μ*Mi-REM potentially more cost-effective in the long-term.

We have demonstrated the utility of our method for constructing different micro-architectures, and illustrated its application for the production of emulsions and microbubbles. Moreover, successful functionalization of PDMS microchannels using hydrophobic silane was performed, indicating that the proposed moulding procedures did not alter the surface chemistry of the PDMS. *μ*Mi-REM could be employed in many applications and laboratory settings as a significantly cheaper and easy-to-implement alternative to conventional, more laborious soft lithographic approaches. It does suffers from limitations in terms of the minimum achievable feature size, which is primarily limited by the micromilling step to ~100 μm (Guckenberger et al. 2015). Furthermore, as the micromilling procedure is usually associated with a certain degree of surface roughness, PMMA smoothing is recommended for those applications requiring high control over the physical properties of the microfluidic inner surfaces. This can be however achieved again using convenient and cost-effective methods (Ogilvie et al. 2010). To facilitate the widespread adoption of our technique, additional information and future developments will be made available online, free of charge.^3^

## Electronic Supplementary Material

ESM 1 (DOCX 356 kb)

ESM 2 (AVI 11427 kb)

## Footnotes

1 If necessary, the microchannel surface can be smoothed by exposure to chloroform vapour as described in Ogilvie et al. (2010).

2 This prediction takes into account the estimated materials’ cost, including PMMA, epoxy resin, PDMS, glass layer and milling tool. It has been estimated that a single milling tool can be employed to fabricate up to 10 devices.

3 www.sonofluidics.co.uk
