# Supplementary material for: Facile and cost-effective production of microscale PDMS architectures using a combined micromilling-replica moulding (μMi-REM) technique
Source: Biomed Microdevices. 2016 Jan 8;18:4. doi: 10.1007/s10544-015-0027-x (PMC4706591; doi:10.1007/s10544-015-0027-x)
Supplement: Supplementary file 1 — (DOCX 356 kb) [file 10544_2015_27_MOESM1_ESM.docx]

**Supplementary Information for:**

**Facile and cost-effective production of microscale PDMS architectures using a combined micromilling-replica moulding (*µ*Mi-REM) technique**

Dario Carugo^1^, Jeong Yu Lee^1^, Anne Pora^1^, Richard J Browning^1^, Lorenzo Capretto^2^, Claudio Nastruzzi^3^ and Eleanor Stride^1,*^

^1^ BUBBL, Institute of Biomedical Engineering, Department of Engineering Science, University of Oxford, Oxford OX3 7DQ, United Kingdom

^2^ School of Pharmacy, University College London (UCL), London WC1E 6BT, United Kingdom

^3^ Department of Life Sciences and Biotechnology, University of Ferrara, Ferrara I-44121, Italy

*Dr Eleanor Stride. Email: Eleanor.stride@eng.ox.ac.uk

S1. Translation of air bubbles within the epoxy layer


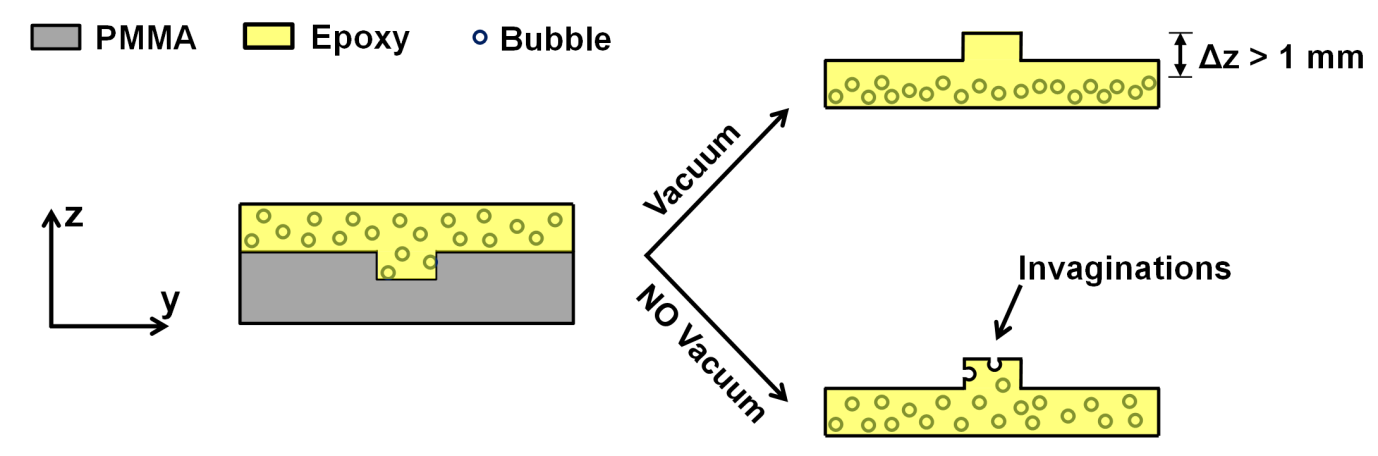


Figure S1 – Schematic depiction of air bubbles’ translation within the liquid bi-component epoxy layer. (left) The mixing of the two components resulted in air bubbles forming within the liquid epoxy. This could potentially limit the effectiveness of the proposed microfabrication process. In order to overcome this limitation, the sample was placed in a vacuum chamber for approximately 4 minutes. As a result of this treatment, the solidified epoxy layer contained a bubble-rich region located away from the microchannel master, and a bubble-free region > 1 mm thick (top-right). If no vacuum is applied, bubbles may remain entrapped within the milled microchannels, resulting in invaginations at the microchannel master surface in the solidified epoxy layer (bottom-right).

S2. Macro- to micro-fluidic connection


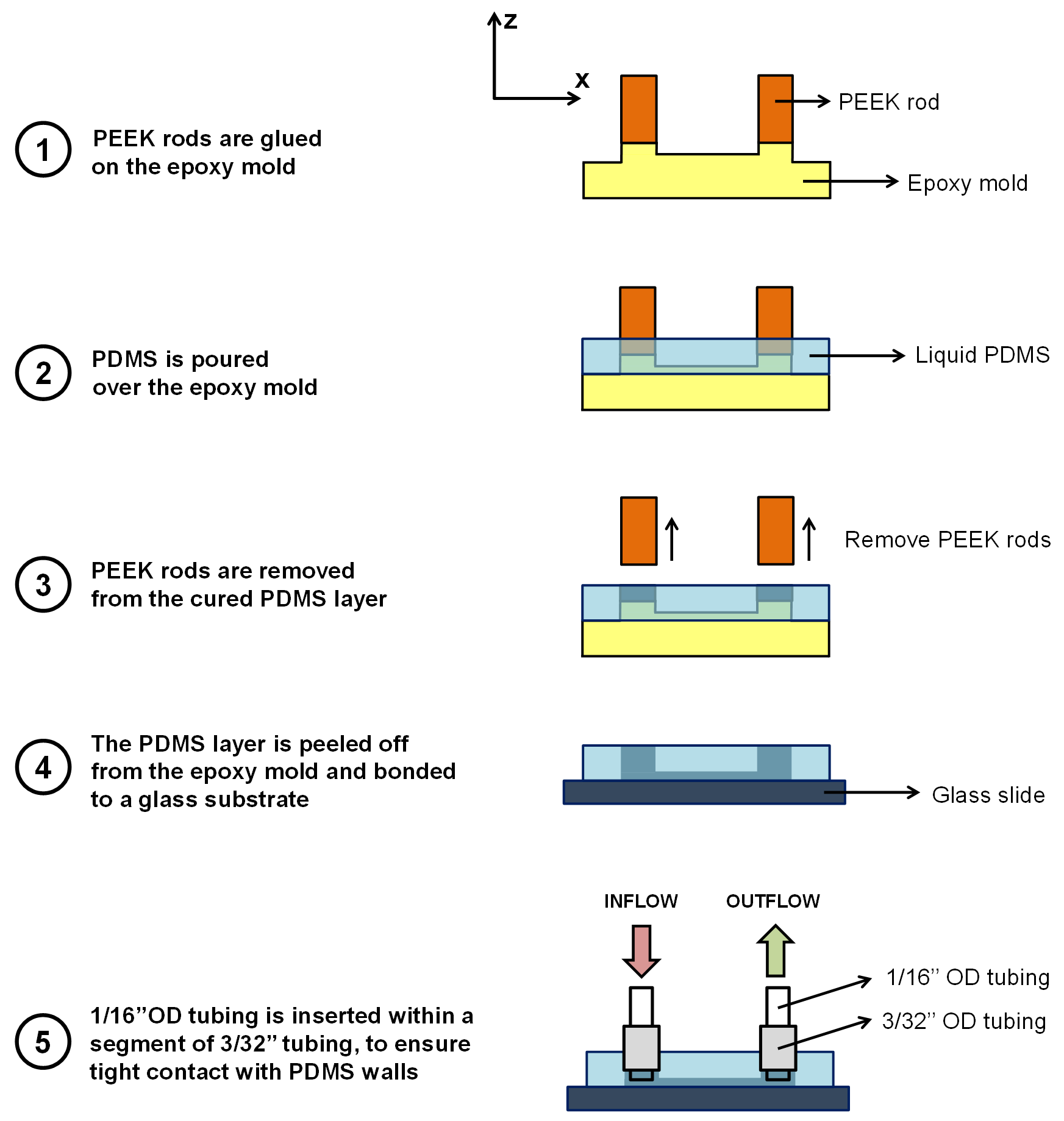


Figure S2 – Schematic depiction of a macro- to micro-fluidic connection strategy adopted in the present study. Note that, depending on the solvents used in the experiment, Tygon® tubing could be directly used as inlet/outlet lines in Step 5, by inserting them within the pre-formed reservoirs. This provides a rapid and convenient strategy for fluid injection and discharge.

S3. Supplementary video illustrating the formation of PBS-in-PLGA emulsions within a ‘T-junction’ microfluidic device fabricated by *µ*Mi-REM. The video has been acquired at a frame rate of 6 frames per second (fps) using a Leica DM500 microscope (Leica Microsystems GmbH, Germany) coupled with a CCD camera (MicroPublisher 3.3 RTV, QImaging, Canada). The microfluidic device corresponds to the one described in Figure 6a. In this specific experiment, volumetric flow rates were set to 0.1 mL/h in IN1 and 0.75 mL/h in IN2.
